# Spt7 Deletion Reveals Vulnerabilities in *Cryptococcus neoformans* Stress Adaptation and Virulence

**DOI:** 10.3390/microorganisms14010095

**Published:** 2026-01-01

**Authors:** Chendi Katherine Yu, Christina J. Stephenson, Benjamin L. Schulz, James A. Fraser

**Affiliations:** School of Chemistry & Molecular Biosciences, Australian Infectious Diseases Research Centre, The University of Queensland, Brisbane, QLD 4072, Australia

**Keywords:** *Cryptococcus neoformans*, SAGA complex, Spt7, virulence

## Abstract

The Spt-Ada-Gcn5 acetyltransferase (SAGA) complex is a conserved transcriptional coactivator that coordinates histone modifications and transcriptional regulation in eukaryotes. In *Cryptococcus neoformans*, SAGA governs key virulence traits, yet the roles of several core scaffold subunits remain undefined. Here, we characterize the functional roles of Spt7, a core SAGA component, in *C. neoformans*. Comparative genomics revealed that *C. neoformans* Spt7 retains conserved histone fold and bromodomain motifs. Deletion of *SPT7* produced pleiotropic phenotypes, including defective melanization and capsule formation, impaired titan cell development, and heightened sensitivity to thermal, metal, antifungal, and cell wall stresses. The *spt7Δ* mutant exhibited strong sensitivity to the echinocandin micafungin, implicating Spt7 in maintaining cell wall integrity. The *spt7Δ* mutant was avirulent in a murine inhalation model. At the chromatin level, *SPT7* deletion disrupted SAGA-dependent histone post-translational modifications, increasing H2B ubiquitination while reducing H3K14ac and H3K18ac levels. Proteomic profiling revealed reduced abundance of ribosomal, mitochondrial, and translational proteins and upregulation of lipid metabolic and secretory pathway components. Collectively, our findings establish Spt7 as a central integrator of SAGA-mediated chromatin regulation, proteomic balance, and virulence in *C. neoformans* and highlight the SAGA core as a potential antifungal target.

## 1. Introduction

Infectious agents rapidly adapt to host environments to establish infection, a process driven by the rewiring of cellular programs aided by epigenetic plasticity, heritable shifts in gene expression independent of DNA mutation. Central to this adaptability are chromatin modifications such as phosphorylation, SUMOylation, and methylation, which dynamically alter histone states and DNA accessibility to influence transcriptional outputs [1]. Functioning as a language of epigenetic signalling, these combinatorial patterns dictate whether genes are activated or silenced in response to stressors like immune attacks or nutrient scarcity [2]. Crucially, these modifications do not act in isolation; they engage in networked interactions where prior modifications recruit or exclude subsequent chromatin-modifying enzymes, creating feedback loops that can amplify or dampen signals [3,4].

Specialized multi-protein complexes that execute context-specific chromatin remodelling are a key component of epigenetic signalling networks. A prime example is the SAGA (Spt-Ada-Gcn5 Acetyltransferase) complex, a conserved transcriptional co-activator critical for regulating gene expression plasticity in the fungal, plant and animal kingdoms [5,6,7]. First characterized in *Saccharomyces cerevisiae* (ScSAGA), this 19-subunit assembly operates through four functional modules that contribute to the dynamic crosstalk of epigenetic modifications. The histone acetyltransferase (HAT) module (Gcn5, Ada2, Ada3, Sgf29) facilitates transcriptional activation by acetylating histones, while the deubiquitinase (DUB) module (Ubp8, Sus1, Sgf73, Sgf11) facilitates transcriptional elongation by removing monoubiquitin. The TF-binding (Tra1) module recruits SAGA to promoter regions by interacting with transcription factors, and the structural core module (Taf5, Taf6, Taf9, Taf10, Taf12, Hfi1, Spt20, Spt7, Spt3, Spt8) stabilizes the complex and facilitates communication with RNA polymerase II [8,9].

Although the modular organization of SAGA is broadly conserved, its subunit composition and domain architecture have diversified substantially across eukaryotic lineages. In *Schizosaccharomyces pombe*, the fission yeast SAGA (SpSAGA) complex retains an identical subunit composition to ScSAGA [10,11]. By contrast, *Homo sapiens* SAGA (HsSAGA) has undergone significant restructuring; it lacks Spt8 but incorporates a splicing module (SF3B3 and SF3B5) [6]; homologs of SF3B3 and SF3B5 exist in *S. cerevisiae* and *S. pombe*, but they do not function as components of the SAGA complex in these species. *Arabidopsis thaliana* SAGA (AtSAGA) diverges further, omitting Spt3, Spt7, and Spt8 while incorporating four plant-specific subunits: SAGA complex subunit 1, 2A and 2B (SCS1, SCS2A, and SCS2B), which are required for normal growth and developmental regulation, as well as TAFL, a TAF-like protein [7].

*SPT7*, originally identified in *S. cerevisiae* through genetic screens for mutations suppressing Ty and δ transposon-induced histidine or lysine auxotrophy, encodes a large, acidic protein critical for SAGA complex integrity in this species [12]. The *S. cerevisiae* Spt7 protein contains two notable structural domains: a central histone fold domain (HFD), which is conserved in the *H. sapiens* and *S. pombe* ortholog SUPT7L, and a bromodomain, which is conserved in the *S. pombe* ortholog but absent in the *H. sapiens* protein. The HFD mediates protein–protein interactions and enables the formation of histone-like heterodimers with other Taf proteins, contributing to the nucleation of a pseudonucleosomal core that stabilizes SAGA [13]. In contrast, the bromodomain recognizes acetylated histone tails and facilitates chromatin interactions [14]. Structural studies in *S. cerevisiae*, including cross-linking mass spectrometry and cryo-EM, show that Spt7 bridges the core and HAT modules, anchoring subunits such as Spt3, Spt8, Ada2, Ada3, Taf5, Taf6, and Taf12, and acting as a central scaffold that stabilizes the overall complex architecture [8,15,16]. Functional analysis of *S. cerevisiae* indicates that Spt7 is essential for normal growth, morphology, and sporulation, underscoring its broad regulatory importance [17].

Full-length Spt7 sequences are highly divergent across eukaryotes, showing only 23.6% identity between *S. cerevisiae* and *S. pombe*, 8.0% between *H. sapiens* and *S. pombe*, and 5.6% between *S. cerevisiae* and *H. sapiens*. However, when the comparison is restricted to HFD, sequence conservation is higher, with 26.5%, 23.5%, and 16.5% identity for the same respective pairs (Supplementary Figure S1). BLAST (https://blast.ncbi.nlm.nih.gov/Blast.cgi, access date: 6 May 2024) searches against plant genomes have identified proteins with partial similarity to the bromodomain of Spt7, but these hits lack full-length homology with Spt7 or its human ortholog SUPT7L, implying that domain-level conservation does not necessarily reflect functional or structural equivalence [18].

While Spt7 has been extensively characterized in *S. cerevisiae*, its role in fungal pathogenesis has only been investigated in the human pathogen *Candida albicans*, where it was shown to regulate morphogenesis, invasiveness and biofilm-related phenotypes and to influence stress responses [19].

SAGA regulates virulence traits such as melanin production, polysaccharide capsule formation, and extracellular enzyme activity in the basidiomycete yeast *Cryptococcus neoformans*, the World Health Organization’s top-ranked critical priority group species on their Fungal Priority Pathogen list [20,21,22,23,24], and mutants lacking *HFI1*, *SPT20*, *GCN5*, or *ADA2* are avirulent in a murine inhalation model of cryptococcosis, whereas deletion of *SGF29* confers hypervirulence [25,26,27,28,29]. Given this, it is important to also understand the role of *SPT7* in this important pathogen.

In this study we identify and characterize *C. neoformans SPT7*, marking it as the third SAGA complex core module gene to be functionally defined in the species. The *spt7Δ* mutant is phenotypically similar to *hfi1Δ* and *spt20Δ* mutants, displaying pleiotropic defects ranging from impaired melanization, reduced capsule size and impaired titan cell development to disrupted secretion of extracellular proteins and heightened sensitivity to stressors. These in vitro defects translate in vivo, with the *spt7Δ* mutant displaying dramatically impaired virulence in a murine model of cryptococcosis. Deletion of Spt7 leads to epigenetic reprogramming through histone modification changes and alters the transcriptional regulation of most SAGA complex components. Proteomic analyses comparing the *C. neoformans hfi1Δ*, *spt20Δ*, and *spt7Δ* mutants revealed lower abundance of ribosomal proteins, translation initiation factors, and mitochondrial respiratory components, alongside increased abundance of lipid biosynthesis and secretory pathway proteins—molecular alterations that mechanistically explain their shared defects in growth, stress tolerance, and virulence. Together, these insights highlight the pivotal role of Spt7 in orchestrating virulence mechanisms and establishing successful infection by this important fungal pathogen of humans.

## 2. Materials and Methods

### 2.1. Bioinformatic Analyses

Bioinformatic analyses were performed largely as described in our previous studies [28,29], with analyses specific to *SPT7* ortholog identification and domain architecture described below. The gene encoding Spt7 in *C. neoformans* was identified via a reciprocal best-hit tBLASTn analysis of the H99O type strain genome [30], using the protein sequence of the *S. cerevisiae* homolog as a query. The identified ORF, *CNAG_03850*, returned Spt7 as a significant hit with lowest E-value in a BLASTp search against the *S. cerevisiae* protein sequence. Comparative sequence alignments of Spt7 from *C. neoformans*, *S. cerevisiae*, *S. pombe*, and *H. sapiens* were conducted using ClustalW v1.4 (MacVector Inc., Apex, NC, USA). Putative Spt7 orthologs were identified across representative eukaryotic species by performing tBLASTn searches using *S. cerevisiae* and *H. sapiens* Spt7 protein sequences as queries against publicly available genome assemblies. Candidate homologs were further assessed based on the presence of conserved domains using the InterPro database v105.0, with emphasis on the HFD (IPR009072) and bromodomain (IPR001487). Species lacking detectable hits under relaxed thresholds or missing domains were classified as Spt7-negative. A taxonomy-based species phylogeny was generated using phyloT v2.

### 2.2. Media and Strains

Media composition, strain handling, and growth conditions were performed as previously described in our related studies [28,29], without further modification. All strains used or generated in this study are listed in Supplementary Table S1. Unless otherwise specified, *C. neoformans* strains were cultivated in liquid yeast peptone dextrose (YPD) medium (2% Bacto Peptone, 1% yeast extract, 2% glucose) or on solid YPD agar plates (YPD medium supplemented with 2% agar), and incubated at 30 °C. *Escherichia coli* strain Mach1 (Thermo Fisher Scientific, Waltham, MA, USA) was grown at 37 °C in lysogeny broth (LB; 1% tryptone, 0.5% yeast extract, 1% sodium chloride) or on LB agar plates (LB with 2% agar), with 100 μg/mL ampicillin added for selection.

### 2.3. Cloning

Cloning strategies were performed as previously described in our related studies [28,29], with gene-specific primers and constructs unique to *SPT7* detailed below. All primers and plasmids used or generated in this study are listed in Supplementary Tables S2 and S3, respectively. Polymerase chain reaction (PCR) amplicons were generated using Phusion High-Fidelity DNA Polymerase (New England Biolabs, Ipswich, MA, USA). The 5′ and 3′ flanking regions of *SPT7* were amplified from *C. neoformans* H99O genomic DNA, while the *NEO* selection marker was obtained from plasmid pJAF1 [31]. Amplicons corresponding to the 1 kb upstream region, the *NEO* marker, and the 1 kb downstream region were assembled into SpeI- and EcoRV-cut pBlueScriptII SK(-) vector using NEBuilder HiFi DNA Assembly (New England Biolabs, USA), resulting in *SPT7* deletion construct pCJS61. To generate a complementation plasmid (pKY09), the *SPT7* locus, including 1 kb of both upstream and downstream flanking sequences, was amplified and cloned into XhoI- and SpeI-cut chromosome 1 Safe Haven vector pSDMA25 [32]. All constructs were validated via Sanger sequencing performed by the Australian Genome Research Facility (AGRF, Melbourne, Australia). Chromatogram data were analyzed using CLC Genomics Workbench v12.1 (QIAGEN, Hilden, Germany).

### 2.4. Creating SPT7 Gene Deletion and Complementation Strains

Generation of gene deletion and complementation strains was performed as previously described [28,29], with modification only to the target locus (*SPT7*). The *spt7Δ* mutant was generated by excising the deletion cassette from plasmid pCJS61 using EcoRV and SpeI (New England Biolabs, USA), followed by biolistic transformation of *C. neoformans* H99 using the W7 hydrochloride method [33,34]. Transformants were selected on YPD agar supplemented with 100 μg/mL G418. To generate the complemented strain, the verified *spt7Δ* mutant was subsequently transformed with the complementation construct pKY09, linearized with PacI (New England Biolabs, USA). Transformants were selected on YPD agar containing 100 μg/mL nourseothricin. Experimental procedures for transformation and verification were identical to those used for constructing the *spt7Δ* strain. Genomic DNA from purified transformants was extracted using the CTAB method [35], digested with appropriate diagnostic restriction enzymes, and resolved on a 1% agarose gel at 24 V overnight. DNA was transferred to Hybond-XL membrane (GE Healthcare, Chicago, IL, USA) for Southern blot analysis [36]. Hybridization was performed overnight at 65 °C using *NEO* or Safe Haven probes labeled with [α-^32^P] dCTP (PerkinElmer, Waltham, MA, USA) using the DECAprime II kit (Thermo Fisher Scientific, USA). Blots were washed with 0.1% SDS, 2× SSC at 65 °C and visualized on a Typhoon PLA 9500 phosphorimager (GE Healthcare, USA).

### 2.5. In Vitro Phenotype Assays

In vitro phenotype assays were conducted as previously described in our related studies [28,29], without further modification. Wild-type (WT), *spt7Δ*, and *spt7Δ* + *SPT7* strains were cultured 16 h in YPD liquid medium, centrifuged and washed twice in distilled water (dH_2_O), and cells resuspended in 1 mL dH_2_O then adjusted to OD_600_ = 1.0. Tenfold serial dilutions were prepared, and 4 µL of each dilution spotted onto yeast nitrogen base (YNB) agar (2% glucose, 10 mM ammonium sulfate) supplemented with 0.5 μg/mL FK506, 0.2 mM NiCl_2_, 5 mM caffeine, 1.5 mM DTT, 0.5 μg/mL amphotericin B, 0.25 mM *tert*-butyl hydroperoxide, 2.5 μg/mL 5-fluorocytosine, 50 μg/mL calcofluor white, 100 μg/mL micafungin or 1% BSA. For virulence trait analysis, 3 µL of a 10^−1^ dilution was spotted onto Christensen’s urea agar [37], l-DOPA agar [38], or egg yolk agar [39]. All spot assays were performed at both 30 °C and 37 °C in biological triplicate. Phenotypes were assessed at 48 and 72 h. Exact concentrations of all chemical stressors used in these assays are specified above and in the corresponding figure panels.

For capsule analysis, strains were cultured in YPD for 24 h and subsequently transferred to RPMI 1640 medium (Life Technologies, Carlsbad, CA, USA) supplemented with 2% glucose and 10% foetal bovine serum. After 48 h of induction, cells were stained with India ink (BD Diagnostics, Franklin Lakes, NJ, USA) and visualized using a Leica DM2500 microscope with a DFC425C camera (Leica, Wetzlar, Germany). Ten images per strain were captured, and measurements of cell and capsule diameters were conducted on 100 cells per strain using Adobe Illustrator. Statistical analysis was performed using Prism 10 (GraphPad Inc., San Diego, CA, USA) via two-tailed unpaired *t*-tests with Welch’s correction.

Titan cell formation was assessed by inoculating 10^7^ cells of each strain into 10 mL YPD medium in T25 Nunclon Delta tissue culture flasks (Thermo Fisher Scientific, USA), followed by 22 h of incubation at 30 °C at 150 rpm. Subsequently, 10^5^ cells were transferred to 1 mL of defined minimal medium (15 mM d-glucose, 10 mM MgSO_4_, 29.4 mM KH_2_PO_4_, 13 mM glycine, 3.0 µM thiamine) and incubated for 120 h at 30 °C with shaking at 800 rpm. Cells were imaged pre- and post-induction using a hemocytometer at 10× and 40× magnification with a Leica DM2500 microscope. Measurements of cell and capsule sizes were performed using Adobe Illustrator and analyzed with Prism 10. Statistical significance was determined using two-tailed unpaired *t*-tests with Welch’s correction.

### 2.6. Murine Inhalation Model of Virulence

The murine inhalation model of cryptococcal infection was performed exactly as previously described in our related studies [28,29], without further modification. Six-week-old female BALB/c mice (Ozgene, Bentley, Australia) were used in a murine inhalation model of cryptococcal infection as previously described [40]. Each strain was tested in a cohort of 10 mice. Mice were anesthetized and inoculated intranasally with a 50 μL droplet containing 5 × 10^5^ *C. neoformans* cells. Animals were housed in IVC cages (maximum 5 mice per cage; Tecniplast, Buguggiate, Italy) with Bed-o’Cobs 1/8″ bedding, Crink-l′Nest nesting material, and cardboard enrichment (The Andersons, Maumee, OH, USA). Mice were provided with ad libitum access to Rat and Mouse Cubes (Specialty Feeds, Glen Forrest, Australia) and water.

Each mouse was weighed and monitored twice daily. Mice exhibiting more than 20% weight loss or signs of severe infection were humanely euthanized by CO_2_ inhalation, with death confirmed by absence of the pedal reflex prior to dissection. Organs (brain, lungs, liver, spleen, kidneys) were collected, weighed, and homogenized using a TissueLyser II (QIAGEN, Germany). Organ homogenates were plated on YPD agar containing 100 μg/mL ampicillin, 50 μg/mL kanamycin, and 25 μg/mL chloramphenicol to determine colony-forming units (CFU) per gram of tissue.

Mice were randomly assigned to experimental groups prior to infection. Animals were monitored daily according to predefined humane endpoint criteria by trained personnel independent of experimental group allocation. Group sizes (*n* = 10 per strain) were selected based on consistency with previously published *C. neoformans* virulence studies of SAGA complex mutants and provide sufficient power to detect survival differences.

Kaplan–Meier survival curves were generated using GraphPad Prism 10 (GraphPad Inc., USA). Statistical significance of survival was assessed using the log-rank test, and organ fungal burdens were compared via one-way ANOVA with Tukey’s multiple comparisons test. A *p*-value < 0.05 was considered statistically significant.

Ethics statement: This study was carried out in strict accordance with the recommendations in the Australian Code of Practice for the Care and Use of Animals for Scientific Purposes by the National Health and Medical Research Council. The protocol was approved by the Molecular Biosciences Animal Ethics Committee of The University of Queensland (AEC approval number: 2022/AE000748). Infection was performed under methoxyflurane anaesthesia, and all efforts were made to minimize suffering through adherence to the Guidelines to Promote the Wellbeing of Animals Used for Scientific Purposes as put forward by the National Health and Medical Research Council.

### 2.7. Western Blot Analyses

Western blot analyses were performed as previously described in our related studies [28,29], without further modification. *C. neoformans* WT, *spt7Δ*, and *spt7Δ* + *SPT7* strains were cultured in YPD liquid medium to an optical density of OD_600_ = 1.0. Cells were harvested by centrifugation and resuspended in 5 mL of Tris-buffered saline (TBS; pH 7.5). Suspensions were aliquoted into 1.5 mL screw-cap tubes (Thermo Fisher Scientific, USA) and snap-frozen in liquid nitrogen. For lysis, a 2× lysis buffer (50 mM Tris-HCl [pH 7.5], 100 mM NaCl, 100 mM PMSF, 1× EDTA-free protease inhibitor cocktail [Roche, Basel, Switzerland]) and approximately 200 µL of 0.5 mm acid-washed silica glass beads (BioSpec Products, Bartlesville, OK, USA) were added to each tube. Cell disruption was carried out using a TissueLyser II (QIAGEN, Germany) at 4 °C for 10 cycles of 90 s of homogenization interspersed with 2 min rest periods. Following homogenization, lysates were transferred to Protein LoBind tubes (Eppendorf, Hamburg, Germany). To stabilize proteins and aid solubilization, glycerol, SDS, DTT, and Triton X-100 were each added separately to each sample to achieve final concentrations of 4%, 1%, 1 mM, and 1%, respectively. Tubes were incubated for 10 min at 4 °C on a HulaMixer Sample Mixer (Thermo Fisher Scientific, USA), then centrifuged at 14,000 rpm for 20 min at 4 °C. The resulting supernatant was collected as the soluble protein extract. Protein concentrations were quantified using the Pierce Detergent Compatible Bradford Assay Kit (Thermo Fisher Scientific, USA) following the manufacturer’s instructions.

Protein extracts (15 ng per sample) were separated on 12% SDS polyacrylamide gels by electrophoresis at 80 V for 3 h. Following separation, proteins were transferred onto Hybond-P PVDF membrane (GE Healthcare, USA) using standard wet-transfer conditions. Membranes were blocked in TBST buffer (25 mM Tris-HCl [pH 7.5], 100 mM NaCl, 0.1% Tween 20) supplemented with 5% bovine serum albumin (BSA), and incubated overnight at 4 °C with rabbit monoclonal primary antibodies, each diluted 1:5000 in TBST with 5% BSA. The following primary antibodies (Cell Signaling Technology, Danvers, MA, USA) were used for detection of histone and histone modification marks: H2B (#12364), H3 (#4499); H2BK120ub (#5546), H3K9ac (#9649), H3K14ac (#7627), H3K18ac (#13998), H4K8ac (#2594), H4K12ac (#13944), H4K16ac (#13534) andH3K4me3 (#9751). Following primary incubation, membranes were washed and probed with HRP-conjugated goat anti-rabbit IgG secondary antibody (#7074; Cell Signaling Technology, USA) diluted 1:1000 in TBST with 5% BSA for 1 h at room temperature. Protein bands were visualized using the SuperSignal^TM^ West Pico PLUS Chemiluminescent Substrate Kit (Thermo Fisher Scientific, USA), and imaged using the ImageQuant 800 GxP biomolecular imager (Cytiva Life Sciences, Marlborough, MA, USA).

### 2.8. RNA Extraction and Reverse Transcription-Quantitative PCR

RNA extraction and RT-qPCR analyses were performed as previously described in our related studies [28,29], with primers targeting SAGA-related genes listed in Supplementary Table S2. Strains were cultured in YPD medium at 30 °C until reaching OD_600_ = 1.0. Cells were harvested, snap-frozen, and lyophilized prior to total RNA extraction using TRIzol reagent (Invitrogen, Waltham, MA, USA). First-strand cDNA synthesis was performed using the SuperScript III First-Strand Synthesis System (Invitrogen, USA) following the manufacturer’s protocol. Quantitative real-time PCR (qRT-PCR) was conducted using SYBR Green Supermix (Applied Biosystems, Waltham, MA, USA) on an Applied Biosystems ViiA 7 Real-Time PCR System. Relative expression levels were calculated using the 2^−ΔΔCT^ method [41], with *ACT1*, *GPD1*, *HHT1*, and *TUB2* used as internal reference genes; *ACT1* was selected for final normalization. Statistical analysis was performed using one-way ANOVA followed by Tukey’s multiple comparisons test, with all data analyzed in GraphPad Prism 10 (GraphPad Software, USA). A *p*-value < 0.05 was considered statistically significant.

### 2.9. Whole Cell Proteomic Analyses

Whole-cell proteomic analyses were performed using a DIA/SWATH-MS workflow established in our laboratory and applied here for comparative analysis of SAGA core mutants. Three biological replicates of each strain (WT, *hfi1Δ*, *spt20Δ* and *spt7Δ*) were cultured in YNB liquid medium at 30 °C and 37 °C and lysed by mechanical disruption as described in the protein extraction protocol above. Lysates were transferred to protein LoBind^®^ tubes (Eppendorf, Enfield, CT, USA) and clarified by centrifugation at 1500 rpm for 1 min. 5 μL of clarified protein was mixed with 50 μL of denaturation buffer (50 mM Tris-HCl [pH 8.0], 6 M guanidine hydrochloride, 10 mM DTT) in protein LoBind^®^ (Eppendorf, USA) tubes and incubated at 30 °C with lateral shaking at 1500 rpm for 30 min. Cysteine alkylation was performed by adding acrylamide to a final concentration of 25 mM and incubation at room temperature for 1 h with shaking. Excess acrylamide was quenched with addition of extra DTT to an additional final concentration of 5 mM. Proteins were precipitated by adding 200 μL (4× volume) of methanol:acetone (1:1) and incubating at −20 °C overnight. Precipitated proteins were pelleted by centrifugation at 18,000× *g* for 10 min, and the supernatant discarded. Pellets were air-dried then resuspended in 50 μL of 50 mM ammonium bicarbonate. Proteomics grade trypsin (0.5 μg per sample, Sigma-Aldrich, St. Louis, MO, USA) was added, and digestion performed overnight at 37 °C with shaking at 1500 rpm. Digested peptides were desalted using C18 ZipTips (Millipore, Burlington, MA, USA), dried using a SpeedVac vacuum concentrator (Thermo Fisher Scientific, USA), and initially solubilized in 7% acetonitrile/0.1% formic acid before being finally resuspended in 20 μL of 0.1% formic acid.

Desalted peptides were analyzed by data-independent acquisition (DIA) LC-ESI-MS/MS on a Prominence NanoLC system (Shimadzu, Tokyo, Japan) coupled to a ZenoTOF mass spectrometer equipped with a Nanospray III interface (SCIEX, Concord, ON, Canada), as previously described [42]. Peptide separation was performed using buffer A (1% acetonitrile, 0.1% formic acid) and buffer B (80% acetonitrile, 0.1% formic acid) with a linear gradient from 10% to 60% buffer B over 24 min. Gas and voltage parameters were adjusted as needed. For DDA, an MS-TOF survey scan from *m*/*z* 350–1800 was acquired for 0.5 s, followed by MS/MS of the top 20 most intense precursor ions (0.05 s per MS/MS). DIA/SWATH-MS was performed under identical LC conditions, using an MS-TOF scan (*m*/*z* 350–1800) for 0.05 s, followed by high-sensitivity DIA with 60 overlapping *m*/*z* isolation windows (1 *m*/*z* overlap), each acquired for 0.025–0.05 s across the *m*/*z* range of 400–1250. CE was automatically assigned by Analyst software v1.7 (SCIEX, Canada) based on window-specific *m*/*z* ranges.

Peptide and protein identification was conducted using ProteinPilot v5.0.1 (SCIEX, Canada). Samples were searched against all predicted proteins from *C. neoformans* (PRJNA411; 7813 proteins, downloaded 28 March 2024). Database were supplemented with known contaminant proteins. Search parameters included: sample type, identification; cysteine alkylation, acrylamide; instrument, ZenoTOF; species *C. neoformans*; ID focus, biological modifications; enzyme, trypsin; and search effort, thorough ID. Quantification of peptide fragments, peptides, and proteins was performed using PeakView v2.2 (SCIEX, Canada) with the following settings: shared peptides allowed; peptide confidence ≥ 99%; FDR ≤ 1%; XIC extraction window, 6 min; XIC width, 75 ppm. Protein-centric analysis and recalculation of protein abundances were performed as previously described [43], applying a strict 1% FDR cutoff [44]. Normalization was conducted relative to either total protein abundance or trypsin self-digest peptides [45]. Quality control and filtering were applied at multiple stages of the DIA/SWATH-MS workflow. Peptide and protein identifications were filtered using a global FDR threshold of 1%, with peptide confidence thresholds set at ≥99%. Only proteins consistently detected across biological replicates were retained for downstream quantitative analysis. Retention time alignment under identical LC conditions and normalization across runs were performed prior to statistical analysis to minimize technical variation. Principal component analysis (PCA) was performed in R (version 4.3.2). Proteins detected exclusively in mutants were designated as uniquely measured in that sample type. For statistical evaluation, PeakView outputs were reformatted as previously described [44], and MSstats v2.4 [46] in R was used to determine significant differences in protein abundance (threshold: *p* ≤ 10^−5^) [47].

## 3. Results

### 3.1. Identification of the Spt7 Ortholog in C. neoformans

To identify the Spt7 ortholog in *C. neoformans*, a tBLASTn search of the H99O genome using *S. cerevisiae* Spt7 as the query was performed, retrieving four candidate loci. Among these, *CNAG_03850*, located on chromosome 2, showed the highest alignment score and query coverage. Importantly, it was the only candidate matching both the histone fold domain (HFD) and the bromodomain. Reciprocal BLAST analysis further confirmed its identity; when *CNAG_03850* was used as a query against the *S. cerevisiae* genome, Spt7 was returned as the top hit, establishing *CNAG_03850* as the *C. neoformans* Spt7 ortholog. In contrast, the remaining three hits aligned only to the bromodomain region and with substantially lower overall similarity. Further reciprocal BLAST analyses revealed that in *S. cerevisiae CNAG_03273* is the ortholog of *TAF2*, *CNAG_05748* the ortholog of *NTO1*, and *CNAG_0066* is the ortholog of *RSC4*.

The *C. neoformans SPT7* gene spans 3575 bp and encodes a predicted 881 residue protein. Its open reading frame contains 13 introns; no introns were identified in the annotated 5′ or 3′ untranslated regions (UTRs), which are 87 bp and 164 bp in length, respectively. The 3′ UTR partially overlaps with the 3′ UTR of downstream gene *CNAG_03849*, which shows highest similarity to *S. cerevisiae* Sip4, a transcriptional activator involved in carbon source regulation. No other structural anomalies were observed.

Protein sequence comparisons revealed modest primary sequence conservation. *C. neoformans* Spt7 shares 13.6% identity (26.9% similarity) with *S. cerevisiae* Spt7, and 8.7% identity (19.1% similarity) with the human ortholog SUPT7L. Even within opisthokonts, divergence is substantial; *S. cerevisiae* Spt7 shares only 6.3% identity (12.5% similarity) with its human counterpart (Supplementary Figure S1). However, this low global similarity contrasts with domain-level conservation. Alignment of the HFD between *C. neoformans* and *S. cerevisiae* reveals 24.8% identity and 48.7% similarity, while the HFD of *C. neoformans* shares 20.5% identity and 35.7% similarity with *H. sapiens* SUPT7L. In addition, the bromodomain shows even higher conservation between *C. neoformans* and *S. cerevisiae*, with 42.9% identity and 59.9% similarity. However, this domain is absent from the human ortholog (Supplementary Figure S1).

**Figure 1:**
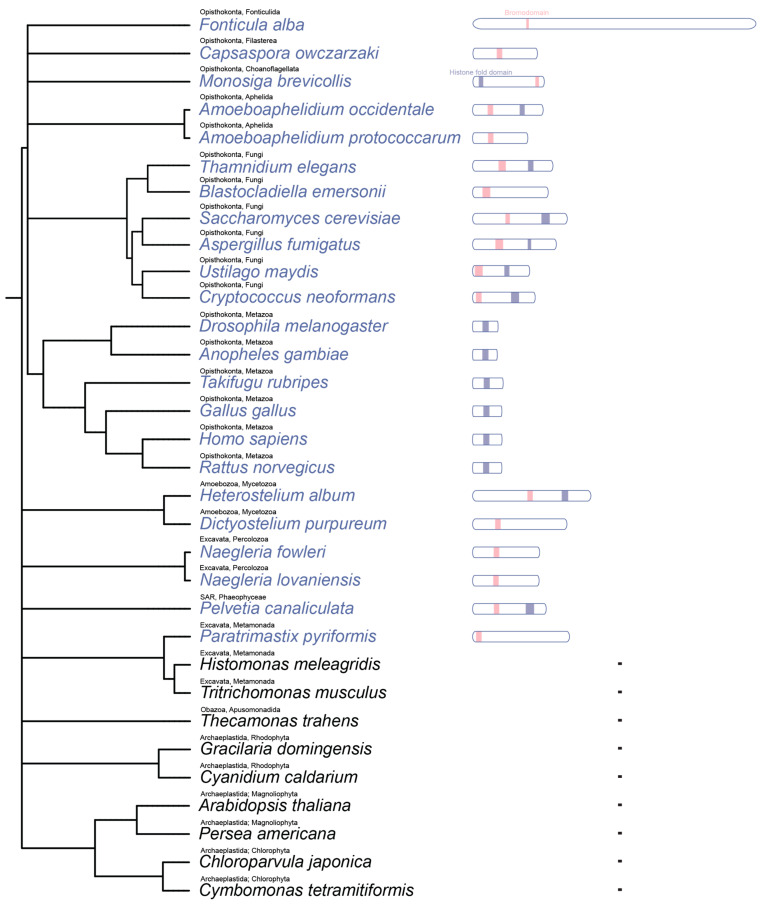
Evolutionary distribution and domain architecture of Spt7-like proteins across representative eukaryotes. A taxonomy-based phylogenetic tree of selected eukaryotic species was generated using phyloT with NCBI Taxonomy IDs as input, to illustrate the distribution of Spt7-like proteins across major eukaryotic lineages. The resulting tree was annotated with key conserved domains when present. Each species is labelled with its assigned supergroup and phylum. Species with detectable Spt7 homologs are shown in blue; those without are shown in black. The presence of the HFD and bromodomain was determined using InterPro annotations, a database that integrates multiple protein family and domain prediction resources.

To further assess the evolutionary distribution of Spt7, we compiled a set of representative eukaryotic species spanning major supergroups, including Opisthokonta, Amoebozoa, Excavata, Obazoa, the SAR clade (Stramenopiles, Alveolates, and Rhizarians), and Archaeplastida, and searched their genomes for Spt7-like proteins using tBLASTn analysis (Figure 1).

The analysis (Figure 1) revealed that Spt7-like proteins with an identifiable HFD are commonly found in fungi and animals, and also occur in several non-metazoan and non-fungal lineages, including *Heterostelium album* (Amoebozoa), *Amoeboaphelidium protococcarum* (an early-diverging opisthokont), and *Pelvetia canaliculata* (SAR). In contrast, while the bromodomain is consistently present in fungi and in several other eukaryotic lineages such as Amoebozoa and SAR, it is not found in Metazoa. No homologs of Spt7 were detected in the Archaeplastida, including land plants (*Arabidopsis thaliana*, *Persea americana*), red algae (*Gracilaria domingensis*), or green algae (*Chloroparvula japonica*).

### 3.2. SPT7 Modulates Stress-Specific Growth Adaptations in C. neoformans

**Figure 2:**
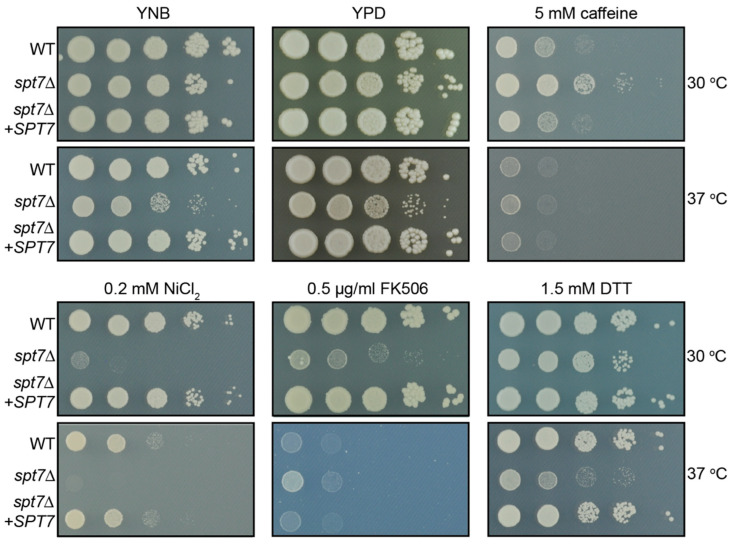
Loss of *SPT7* results in stress-associated phenotypes. Growth of 10-fold serial dilutions of WT, *spt7Δ*, and *spt7Δ* + *SPT7* strains of *C. neoformans* on a variety of media. Pictures were taken after 48 h of growth.

To assess whether Spt7 influences stress adaptation in *C. neoformans*, we constructed a *spt7Δ* mutant and complemented strain, and examined their growth phenotype under a range of conditions. On nutrient-rich complete (YPD) and defined (YNB) media at 37 °C, the *spt7Δ* strain exhibited weaker growth than WT and complemented (*spt7Δ* + *SPT7*) strains (Figure 2).

**Figure 3:**
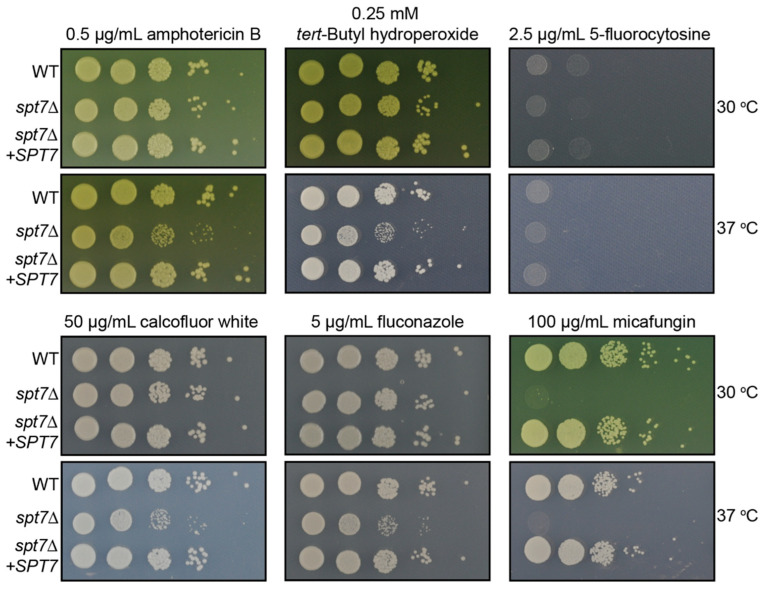
Loss of *SPT7* confers sensitivity to multiple stress conditions, including antifungal, oxidative, and cell wall stress. Growth of 10-fold serial dilutions of WT, *spt7Δ*, and *spt7Δ* + *SPT7* strains of *C. neoformans* on a variety of media. Pictures were taken after 48 h of growth.

The response of the *spt7Δ* mutant to specific chemical stressors also differed from those of WT and complemented strains. Growth was significantly impaired upon exposure to 0.2 mM nickel chloride at both 30 and 37 °C, while treatment with 5 mM caffeine (a known cell wall perturbagen) paradoxically enhanced growth in the mutant at 30 °C. Sensitivity of this mutant to the calcineurin inhibitor FK506 (tacrolimus) was elevated at 30 °C and only slightly increased at 37 °C relative to WT. The mutant also displayed reduced growth in response to the presence of calcofluor white (cell wall stress), 1.5 mM dithiothreitol (oxidative stress) and tert-butyl hydroperoxide (oxidative stress) at 37 °C (Figure 2 and Figure 3).

In clinical settings, *C. neoformans* encounters antifungal pressure during induction (amphotericin B and flucytosine) and maintenance (fluconazole) therapy. The *spt7Δ* mutant increased sensitivity to amphotericin B but not to 5-fluorocytosine compared to WT at 37 °C (Figure 3). However, micafungin, an echinocandin typically ineffective against *C. neoformans*, now impaired *spt7Δ* growth at both 30 °C and 37 °C (Figure 3).

### 3.3. SPT7 Orchestrates Divergent Modulation of C. neoformans Virulence Traits In Vitro

**Figure 4:**
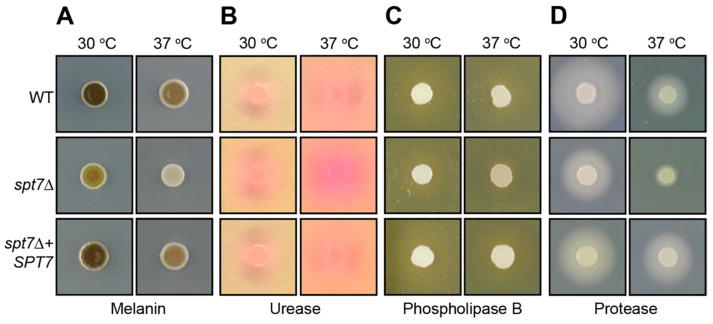
Loss of *SPT7* affects the production of *C. neoformans* virulence factors in vitro. Production of laccase was determined on l-DOPA agar (**A**), urease on Christensen’s urea agar (**B**), phospholipase B on egg yolk agar (**C**) and protease on 2% glucose YNB agar supplemented with 0.1% bovine serum albumin (**D**).

Deletion of *SPT7* in *C. neoformans* leads to differential regulation of key virulence factors under laboratory conditions. The *spt7Δ* mutant showed diminished production of melanin (_L_-DOPA agar), extracellular proteases (BSA agar), and phospholipase B (egg yolk agar). In contrast, urease activity (Christensen’s agar) was significantly increased compared to WT (Figure 4).

**Figure 5:**
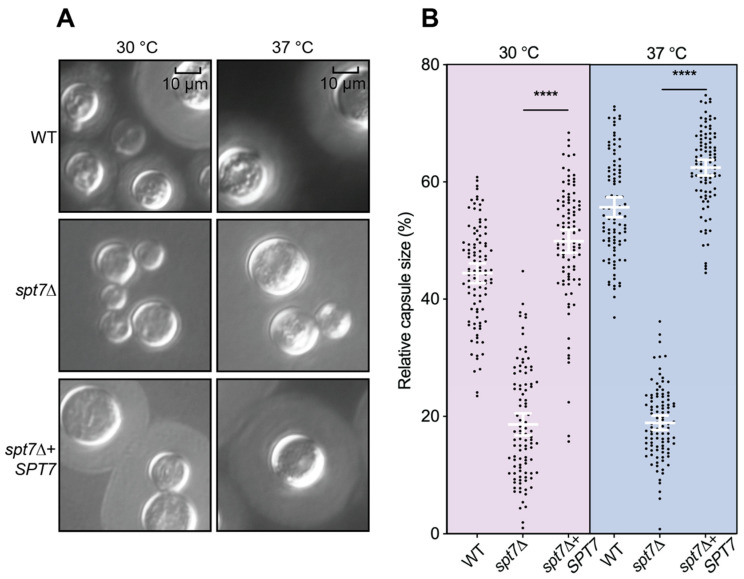
The *sp7Δ* mutant has a capsule defect. (**A**) Strains were incubated in RPMI 1640 medium supplied with 10% foetal bovine serum at 30 °Cand 37 °C. At 48 h, cells were stained with India ink. (**B**) Relative capsule size of 100 cells from WT, *spt7Δ* and *spt7Δ* + *SPT7*. Strains were compared by two-tailed *t*-tests (unpaired) with Welch’s correction. Error bars show the means ± 95% confidence intervals, *n* = 100. **** represents significant difference (*p* < 0.0001).

Assessment of capsule production in the *spt7Δ* mutant grown in RPMI 1640 medium and visualized by India ink staining revealed an easily observed strong reduction in capsule size compared to WT and complemented strains. Quantitative measurements confirmed a statistically significant decrease in capsule dimensions (*p* < 0.0001), consistently observed at both tested temperatures (Figure 5).

**Figure 6:**
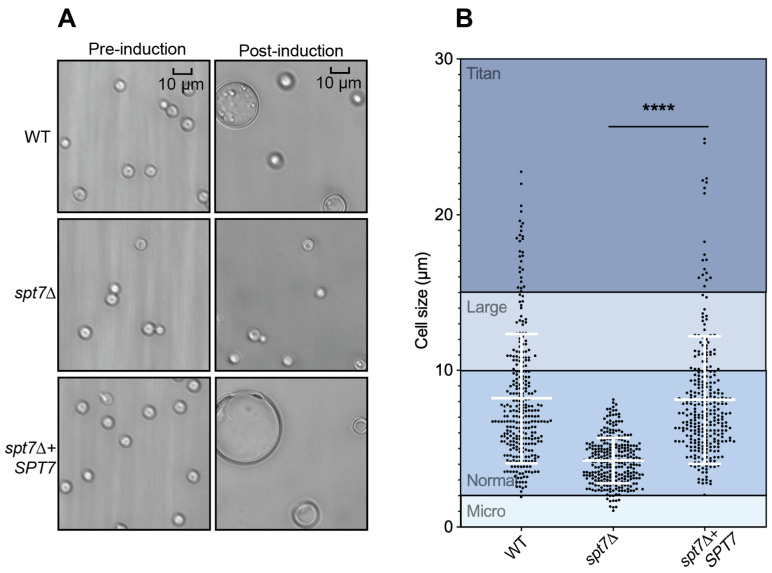
The *spt7Δ* mutant does not form titan cells. (**A**) 105 cell number for each strain were incubated in minimal medium at 30 °C. Images were captured before and after 5 days incubation. (**B**) Cell sizes (without capsule) of 300 cells from WT, *spt7Δ* and *spt7Δ* + *SPT7*. Strains were compared by two-tailed *t*-tests (unpaired) with Welch’s correction. Error bars show the means ± 95% confidence intervals, *n* = 300. **** represents significant difference (*p* < 0.0001).

The *spt7Δ* mutant was unable to form titan cells, which is a key virulence-associated morphological adaptation, and this defect was fully rescued by reintroduction of the *SPT7* allele (Figure 6A). To quantify the phenotype, cell body diameters (excluding capsules) were measured in 300 cells per strain. Morphometric analysis revealed a highly significant reduction in cell size in the *spt7Δ* mutant compared to WT (*p* < 0.0001, Figure 6B).

### 3.4. SPT7 Governs C. neoformans Virulence in a Murine Inhalation Model

**Figure 7:**
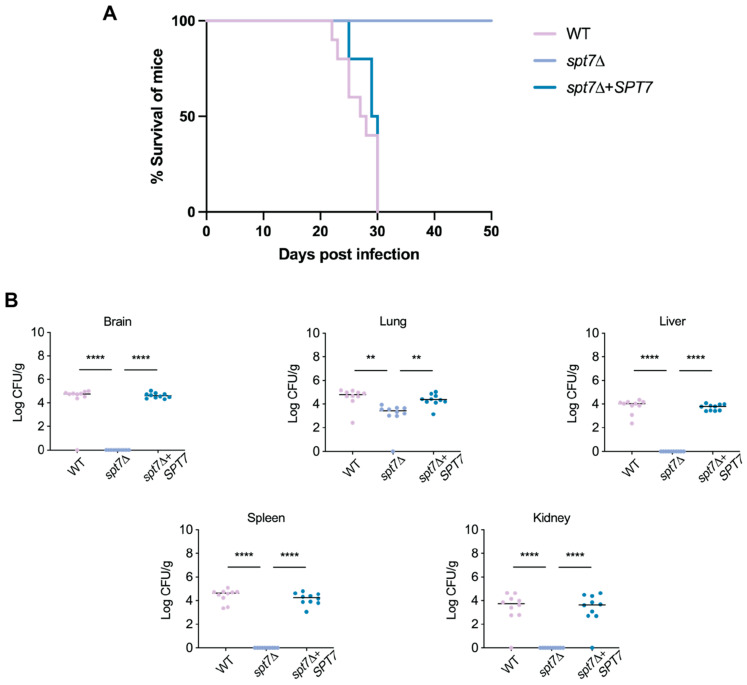
*SPT7* is required for virulence of *C. neoformans* in a murine inhalation model of infection. (**A**) Six-week-old mice were subjected to intranasal infection with either 105 cells of the WT strain, the *spt7Δ* mutant strain, or the *spt7Δ* + *SPT7* complemented strain. Kaplan–Meier survival curves were generated, and statistical significance was determined using log-rank tests. All mice infected with the *spt7Δ* mutant strain survived, and there was no statistically significant difference in survival between mice infected with the WT strain and those infected with the *spt7Δ* + *SPT7* complemented strain (*p*-value > 0.05). (**B**) Fungal organ burden in the infected mice was assessed for WT, *spt7Δ*, and *spt7Δ* + *SPT7* strains. Statistical significance was determined using one-way ANOVA with Tukey’s multiple comparisons test. *p*-values of <0.05 were considered significant. Median is represented by a black line. Fungal burden in mice infected with the WT strain or the *spt7Δ* + *SPT7* complemented strain was significant higher (** *p*-value < 0.01, **** *p*-value < 0.0001) compared to mice infected with the *spt7Δ* mutant strain.

Building on evidence of pivotal role of Spt7 in stress adaptation and virulence factor regulation in vitro, we evaluated its contribution to pathogenicity using a murine inhalation model. Mice infected with WT or complemented (*spt7Δ* + *SPT7*) strains began to developed disease symptoms by day 22, requiring euthanasia by week four. In the contrast, mice infected with the *spt7Δ* mutant remained asymptomatic for 50 days, exhibited steady weight gain, and showed no clinical signs of disease (Figure 7A). While post-mortem analysis revealed no detectable fungal burden in the liver, spleen or kidneys of *spt7Δ*-infected animals, residual *C. neoformans* cells were recovered from the lungs of nine mice, similar to the attenuation observed for the other SAGA core mutants *hfi1Δ* and *spt20Δ* [28,29] (Figure 7B).

### 3.5. SPT7 Orchestrates SAGA-Dependent Histone Modification Landscapes in C. neoformans

**Figure 8:**
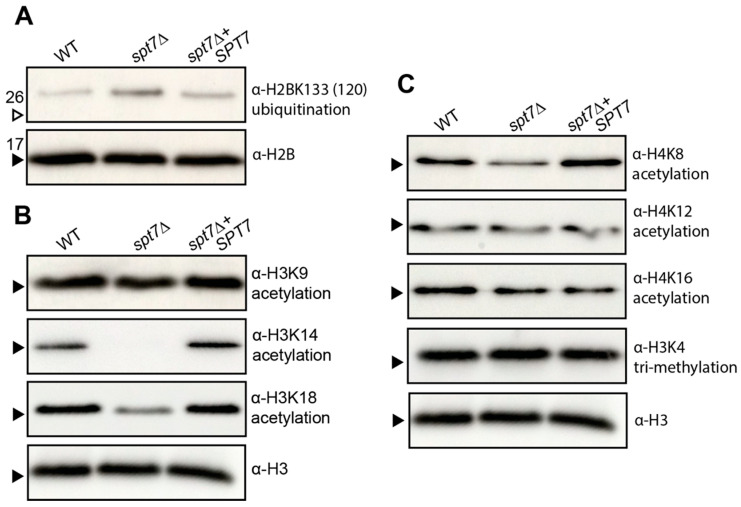
Spt7 influences histone deubiquitination and acetylation in *C. neoformans*. (**A**) Ubiquitination of histone 2B was detected using anti-H2BK120ub. (**B**) Acetylation of histone H3 was detected using anti-H3K9ac, -H3K14ac, and -H3K18ac antibodies. (**C**) Acetylation of histone H4 and methylation of H3 was detected using anti-H4K8ac, -H4K12ac, -H4K16ac, and -H3K4trimethyl antibodies. Arrows indicate the 17 kDa (black arrow) and 26 kDa (white arrow) bands from the Broad Range Color Prestained Protein Standard (NEB).

Our previous studies demonstrated that Hfi1 and Spt20 are required for coordinating post-translational histone modification functions of the SAGA complex [28,29]. To determine whether Spt7 affects these epigenetic marks, we also assessed the impact of *SPT7* deletion on these key epigenetic marks. Western blot analysis revealed the *spt7Δ* mutant exhibited increased H2BK133 ubiquitination (Figure 8A) and decreased or absent acetylation at H3K14, H3K18 and H4K8 (Figure 8B,C), while levels of H3K9ac, H4K12ac, H4K16ac, and H3K4me3 remained unchanged (Figure 8C). The increased H2B ubiquitination and reduced H3K14ac levels largely mirror those observed in *hfi1Δ* and *spt20Δ* mutants, while the decrease in H4K8ac and loss of H3K14ac appears specific to *spt7Δ* [28,29].

### 3.6. SPT7 Deletion Alters SAGA Complex Gene Expression

**Figure 9:**
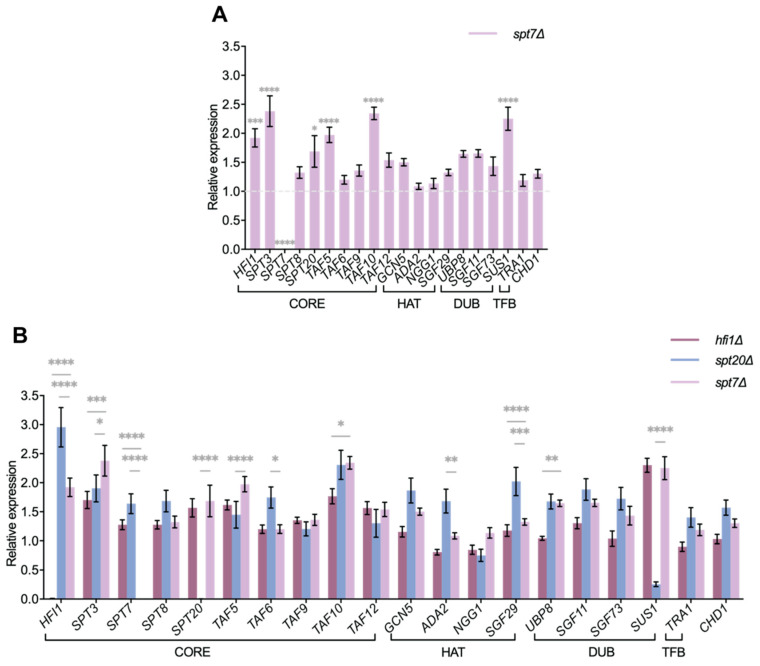
Loss of *SPT7* affects the transcript levels of SAGA protein-encoding genes more significantly compared to *hfi1Δ* and *spt20Δ*. (**A**) The influence of the *spt7Δ* mutation on the abundance transcripts encoding components of the SAGA complex. (**B**) A comparison of the transcript levels of SAGA protein-encoding genes between *spt7Δ*, *spt20Δ* and *hfi1Δ*. *ACT1* was used as the control for normalization. The relative expression for WT genes is 1, represented by a grey dashed line. Error bars illustrate the standard errors computed from three biological replicates, each with three independent technical replicates, while the asterisks indicate significant differences (**** *p*-value ≤ 0.0001; *** *p*-value < 0.001; ** *p*-value < 0.01; * *p*-value ≤ 0.05), as determined by one-way ANOVA with Tukey’s multiple comparisons test.

To investigate the loss of *SPT7* on SAGA complex function, we performed RT-qPCR to quantify transcript levels of SAGA-associated genes, comparing the *spt7Δ* mutant to previously characterized *hfi1Δ* and *spt20Δ* strains. Despite overlapping phenotypic traits, the three mutants exhibited distinct transcriptional profiles. Similarly to the *hfi1Δ* mutant but differently from *spt20Δ*, the *spt7Δ* mutant exhibited transcriptional upregulation across two SAGA modules, mainly in the core module (*SPT3*, *SPT20*, *HFI1*, *TAF5*, *TAF10*), and one element of DUB (*SUS1*) (Figure 9A). In addition, *GCN5* and *UBP8*, encoding key histone-modifying enzymes, were significantly upregulated in *spt20Δ* (*p* < 0.05), a pattern not observed in either the *spt7Δ* or *hfi1Δ* mutant (Figure 9B).

### 3.7. Comparative Proteomic Profiling Reveals the Impact of SAGA Core Module Subunit Deletions

**Figure 10:**
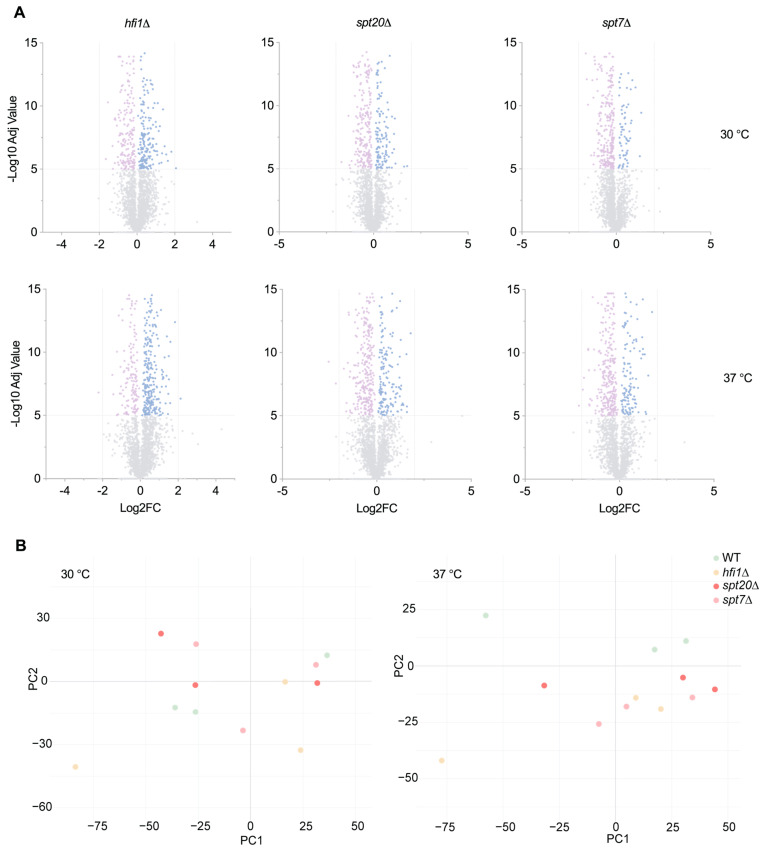
Shared proteomic signature of SAGA-core deletion mutants. (**A**) A volcano plot shows the Log_2_-fold change (Log2FC) versus −log_10_ (adjusted *p*-value) for each protein quantified in the core mutant strains grown at 30°C or 37 °C. Grey dots represent non-significant changes, while coloured dots highlight proteins significantly altered compared to WT (adjusted *p*-value < 10^−5^): pink for downregulated and blue for upregulated proteins. (**B**) PCA of protein abundance normalized to the abundance of trypsin in each sample (biological replicates). The first component (*x*-axis) accounted for 32.3% (30 °C) and 30.2% (37 °C) of the total variance, and the second (*y*-axis) accounted for 6.4% (30 °C) and 5.6% (37 °C).

To better understand how the SAGA complex contributes to global protein homeostasis, we performed label-free data-independent acquisition (DIA/SWATH-MS) quantitative proteomic analysis on deletion mutants lacking key subunits of the SAGA core module (*hfi1Δ*, *spt7Δ* and *spt20Δ*) under basal (30 °C) and stress (37 °C) conditions. Out of around 3200 proteins reproducibly quantified across all samples, fewer than 20% met the differential abundance threshold (*p*-value < 10^−5^) in any single mutant, yielding largely symmetrical volcano plots (Figure 10A). PCA of the proteomic variance revealed that the proteomes of mutants and WT separated at 37 °C, but not at 30 °C (Figure 10B).

A significant downregulation of ribosomal proteins was observed across all three mutants (Supplementary Tables S4–S11). Components of both the 40S (including S3, S7, S10e, S17, S26, and mS29) and 60S (L2, L3, L13, L22e, L32e, and L36) subunits were consistently reduced in abundance, representing approximately 15% of the ~80 ribosomal proteins encoded in the *C. neoformans* genome [48]. Consistent with a general suppression of protein synthesis pathways, the expression of serine/threonine-protein kinase TOR was markedly reduced in all mutants, particularly at 37 °C. Additional reductions were found in aminoacyl-tRNA synthetases, including arginine-, lysine-, and aspartate-tRNA ligases most of which correspond to the cytoplasmic isoforms responsible for translation of nuclear-encoded proteins. Mitochondrial dysfunction was also evident from the proteomic data. All three mutants exhibited decreased levels of proteins involved in oxidative phosphorylation, such as succinate dehydrogenase subunits (complex II) and ATP synthase components. Perturbations in the mitochondrial contact site and cristae organizing system (MICOS) complex, which maintains mitochondrial inner membrane architecture, were also observed, particularly at higher temperatures. Interestingly, several proteins involved in membrane lipid metabolism and proteostasis were upregulated. Although squalene synthase, a key enzyme in the early steps of ergosterol biosynthesis, was upregulated in all three mutants, downstream enzymes in the pathway were not similarly elevated, suggesting a metabolic bottleneck rather than enhanced sterol production. This imbalance likely contributes to the increased amphotericin B sensitivity observed in these strains (Figure 3). Furthermore, expression of the Golgi-resident protease kexin was significantly elevated at 37 °C in each mutant. While all three SAGA core mutants shared a global reduction in ribosomal and translation-associated proteins, principal component and differential abundance analyses revealed distinct proteomic signatures for each strain, indicating that the loss of individual SAGA subunits elicits overlapping but non-identical effects on the cellular proteome.

## 4. Discussion

Across eukaryotes, the SAGA complex functions as a crucial transcriptional coactivator, particularly in the regulation of genes responsive to environmental stress. In *S. cerevisiae*, this complex is organized into four modules: HAT, DUB, TF-binding, and a central core module that serves as a structural scaffold. Our earlier work in *C. neoformans* centred on Hfi1 and Spt20, both of which are components of the core module [28,29]. Building on this foundation, we now shift our attention to another integral core protein, Spt7, to further elucidate the architecture and function of the *C. neoformans* SAGA complex.

Our phylogenetic and domain-based survey of Spt7 across representative eukaryotes reveals a mosaic pattern of conservation, highlighting both the ancient origin and lineage-specific remodelling of this essential SAGA subunit. The complete absence of Spt7 orthologs in Archaeplastida and certain basal eukaryotes suggests lineages suggests that this subunit either emerged after the divergence of these groups or was secondarily lost multiple times during eukaryotic evolution. The bromodomain of Spt7 shows a unique distribution; metazoan Spt7 orthologs entirely lack the bromodomain, pointing to possible functional replacement or reduced reliance on histone acetylation-mediated chromatin anchoring within animal SAGA complexes. Several species retain a bromodomain without a detectable HFD, suggesting possible domain shuffling or repurposing of bromodomain-containing proteins independent of canonical Spt7 scaffolding roles. These findings support a model in which Spt7 originated early in eukaryotic evolution but diversified structurally and functionally in response to lineage-specific regulatory contexts.

We deleted the *SPT7* gene in *C. neoformans*, revealing that the mutant exhibits multiple stress-related phenotypes, underscoring the pleiotropic role of the genes encoding SAGA core proteins. Similarly to *hfi1Δ* and *spt20Δ*, these phenotypes included resistance to caffeine, heightened sensitivity to NiCl_2_, and a temperature-dependent FK506 response, suggesting involvement in oxidative stress response, calcineurin signalling, and metal ion homeostasis, respectively [49,50,51,52]. Although the *spt7Δ* strain showed reduced growth on plates containing *tert*-butyl hydroperoxide or calcofluor white, these effects were not markedly different from its already diminished baseline growth on standard YNB medium (Figure 2). Accordingly, this likely reflects an intrinsic growth defect rather than specific sensitivity to these stressors. In contrast to the pronounced responses observed under other conditions, these findings suggest that Spt7 is not broadly required for ER stress tolerance, ROS defence, or chitin remodelling [53,54,55,56,57]. Collectively, these data support a model in which Spt7 contributes selectively to cell wall maintenance and certain stress-response pathways, rather than functioning as a universal regulator of stress resistance.

Standard clinical treatment for cryptococcal meningoencephalitis typically involves amphotericin B, which binds to ergosterol in the fungal membrane; flucytosine, a prodrug activated via the pyrimidine salvage pathway; and fluconazole, which inhibits a key step in ergosterol biosynthesis. The *spt7Δ* mutant exhibited increased sensitivity to amphotericin B and fluconazole at 37 °C, whereas its response to flucytosine was comparable to the WT. However, because the mutant already displays slower baseline growth on non-stress media (YNB), these apparent drug sensitivities likely reflect an intrinsic growth defect rather than specific hypersensitivity to these antifungals. Accordingly, we do not interpret these results as evidence of bona fide antifungal drug hypersensitivity.

Despite the intrinsic resistance of *C. neoformans* to echinocandins, antifungal agents that target β-1,3-glucan synthase, and compromise cell wall integrity. Consistent with our previous findings that loss of *SPT20* compromises cell wall integrity and increases micafungin sensitivity, the *spt7Δ* mutant also displayed strong sensitivity to micafungin at both 30 °C and 37 °C [29]. These findings suggest that Spt7 contributes to fungal cell wall integrity through previously unrecognized mechanisms [58,59]. Typically, *C. neoformans* resists micafungin due to its unique cell wall structure, characterized by reduced β-1,3-glucan levels compared to echinocandin-susceptible fungi [60]. The unexpected micafungin sensitivity in the spt7Δ strain suggests that additional factors beyond β-1,3-glucan content contribute to echinocandin resistance, revealing a potential vulnerability. These results raise the possibility that perturbation of Spt7 function could sensitize *C. neoformans* to echinocandins, suggesting a potential avenue for future combination therapies against cryptococcal infection.

The *spt7Δ* mutant exhibited markedly reduced virulence in a murine inhalation model of cryptococcal infection. Over the 50-day observation period, mice infected with *spt7Δ* showed no signs of disease. This outcome, reminiscent of the response observed in *hfi1Δ*- or *spt20Δ*-infected mice, indicates that loss of *SPT7* severely compromises the ability of *C. neoformans* to cause disease. Although residual fungal cells may persist in the lungs, the mutant fails to establish progressive or symptomatic infection, underscoring the critical role of Spt7 in full virulence expression.

The attenuated virulence observed in the *spt7Δ* mutant highlights the essential role of Spt7 in sustaining the pathogenic potential of *C. neoformans*. The reduction in virulence aligns with the broad spectrum of in vitro phenotypes exhibited by the mutant, many of which suggest defects in stress adaptation and regulatory signalling. Like *hfi1Δ* and *spt20Δ*, deletion of *SPT7* disrupts multiple virulence-associated processes, likely through its central role in maintaining the structure and function of the SAGA coactivator complex. At the chromatin level, *spt7Δ* significantly impairs several histone PTMs, including acetylation of H3K14 and H3K18, as well as deubiquitination of H2B, modifications also affected in the *hfi1Δ* and *spt20Δ*. However, a key difference is that *spt7Δ* reduces acetylation at H4K8, a PTM not impacted by the loss of Hfi1 or Spt20, suggesting that Spt7 exerts a distinct and possibly expanded regulatory influence within the SAGA complex, potentially linking it to additional chromatin-modifying pathways beyond those mediated by the other core scaffold subunits.

Transcriptomic profiling revealed a distinction between the three core mutants: *spt20Δ* altered the expression of 15 SAGA-related genes, compared to only 6 in *hfi1Δ* or *spt7Δ*. This pattern suggests that Spt7 plays a role comparable to Hfi1, though less extensive than Spt20, in maintaining SAGA complex stability and coordinating global transcriptional regulation. Differences in the extent and specificity of SAGA gene disruption may explain the phenotypic divergence between the three mutants, whereas overlapping changes likely drive their shared defects in stress tolerance and virulence attenuation.

The proteomic alterations identified in *hfi1Δ*, *spt7Δ*, and s*pt20Δ* mutants indicate broad disruptions in multiple cellular processes, with several trends shared across the mutants. The broad downregulation of ribosomal proteins and translation initiation factors offers a direct explanation for the reduced growth rates and temperature sensitivity of these strains. Given the role of TOR signalling in coordinating ribosome biogenesis with environmental inputs, the decreased TOR expression further supports a model of global translational repression in these mutants. Such repression would severely limit their capacity to respond to stress or proliferate under host-like conditions [61]. The increased sensitivity to oxidative stress agents such as DTT and tert-butyl hydroperoxide across all three mutants aligns with the observed downregulation of mitochondrial respiratory proteins. Impaired oxidative phosphorylation and mitochondrial membrane organization would directly compromise ATP production and redox balance, which are critical under oxidative challenge [62]. The reduction in MICOS complex proteins further indicates disruption of mitochondrial integrity, which could contribute to both energetic deficits and increased cell death under stress.

Mitochondrial function is closely integrated with cellular metabolic and membrane-associated processes that could support adaptation to stress conditions. Disruption of mitochondrial integrity in the *spt7Δ* mutant may therefore trigger compensatory remodeling of lipid biosynthetic pathways in an attempt to preserve membrane stability and cellular viability. In this context, the observed upregulation of lipid metabolism-associated proteins likely reflects a secondary response to impaired mitochondrial function rather than a primary defect in lipid regulation.

Evidence from both phenotypic and proteomic analyses supports the presence of membrane-associated defects. All three mutants displayed altered responses to membrane stressors, including unexpected susceptibility to micafungin. The increased expression of squalene synthase likely reflects a compensatory response aimed at reinforcing membrane lipid biosynthesis [63,64]. However, this response may be insufficient or misregulated, contributing to the increased vulnerability to cell wall- and membrane-targeting agents such as caffeine and echinocandins. The strong upregulation of kexin protease at 37 °C in all three mutants suggests an adaptive response aimed at managing protein secretion or alleviating ER-Golgi proteostasis stress [65,66]. However, the reduced production of extracellular virulence factors such as phospholipase B, proteases, and melanin implies that this compensatory mechanism is inadequate. This conclusion is further emphasized by the severe reduction in capsule size and the complete failure to form titan cells, phenotypes that depend on tightly regulated protein expression and secretion pathways. The coupling of translational defects with disrupted protein folding and secretion likely accounts for the global attenuation of virulence factor expression in these mutants. Taken together, these results highlight the essential role of the SAGA complex, through Hfi1, Spt7, and Spt20, in coordinating transcriptional programs that sustain core metabolic and stress response networks. Loss of these components leads to a breakdown in translational capacity, mitochondrial function, and membrane stability, resulting in impaired adaptation to host-relevant conditions and diminished pathogenic potential.

Although our analysis focused primarily on SAGA component genes, the influence of Spt7 likely extends far beyond this subset. Through its role in chromatin modification, Spt7 is likely to contribute to widespread transcriptional and epigenetic remodelling. Future transcriptomic studies will be essential for delineating these broader effects and to map the full regulatory landscape governed by the SAGA complex. Such investigations promise to uncover new dimensions of fungal pathogenesis and expand our understanding of co-transcriptional regulation in eukaryotic pathogens.

Our findings are consistent with studies in *S. cerevisiae*, where Spt7 is essential for proper SAGA assembly and function [67]. In *S. cerevisiae*, the role of Spt7 as a core structural component of the SAGA complex is well-established, particularly from high-resolution cryo-EM studies [68]. Within this architecture, Spt7 is embedded in the core module, forming residue-level interactions with several subunits, including Ada2, Ada3, Taf5, Taf6, Taf10, Taf12, Spt3 and Spt8. These contacts highlight its central bridging function within the complex. Deletion of *SPT7* in *S. cerevisiae* result in significant disruptions of SAGA structural integrity and DUB module attachment [67,69,70]. In *C. neoformans*, our data indicate that deletion of these same core genes does not severely impact growth under standard conditions but causes pronounced defects under stress, suggesting a condition-dependent requirement for complex integrity. These observations point to a conserved yet contextually flexible role for the core module across divergent fungi. However, the exact structural consequences of core subunit deletions remain undefined. Direct structural analysis of mutant SAGA complexes in either species would be essential to resolve how core subunits contribute to the modular stability and functional adaptability of this key transcriptional co-activator.

Chromatin regulation in eukaryotes involves a broader landscape of remodelling complexes. Polycomb repressive complexes (PRC) and chromatin assembly factor-1 (CAF-1), for instance, modulate gene expression by repressing transcription or facilitating nucleosome assembly in response to environmental signals [71,72]. In contrast, SAGA acts as a coactivator complex that dynamically promotes transcription, integrating chromatin modification with stress-responsive gene expression. What distinguishes *C. neoformans*, however, is the unique role SAGA appears to play in regulating both classical virulence traits and stress adaptation mechanisms crucial for host survival. The disruption of *SPT7* not only compromises these regulatory pathways but also renders the pathogen sensitive to micafungin, an antifungal to which *C. neoformans* is typically resistant. The unexpected vulnerability to micafungin, coupled with impaired virulence and stress tolerance, positions the SAGA complex as a compelling target for antifungal drug development. Future exploration of specialization of SAGA in *C. neoformans* may uncover novel strategies to disrupt fungal pathogenesis and enhance treatment efficacy.

## Data Availability

The mass spectrometry proteomic data have been deposited to the ProteomeXchange Consortium via the PRIDE [73] partner repository with the data set identifier: PXD071194.

## Supplementary Materials

The following supporting information can be downloaded at: https://www.mdpi.com/article/10.3390/microorganisms14010095/s1. Figure S1: (A) Protein alignments of full length Spt7 from *S. cerevisiae*, *S. pombe*, *C. neoformans* and *H. sapiens*. (B) Protein alignments of HFD of Spt7 from *S. cerevisiae*, *S. pombe*, *C. neoformans* and *H. sapiens*. (C) Protein alignments of Bromo domain of Spt7 from *S. cerevisiae*, *S. pombe* and *C. neoformans*; Table S1: Strains used in this study; Table S2: Primers used in this study. SAGA complex gene-specific RT-PCR primers were designed to span exon-exon junctions, ensuring amplification of cDNA rather than genomic DNA; Table S3: Plasmids used in this study; Table S4A: Proteins consistently differentially expressed in *hfi1*Δ, *spt7*Δ and *spt20*Δ mutants at 30 °C; Table S4B: Proteins consistently differentially expressed in *hfi1*Δ, *spt7*Δ and *spt20*Δ mutants at 37 °C; Table S5-S11: Raw and processed quantitative proteomic data from DIA/SWATH-MS analysis of WT, *hfi1Δ*, *spt20Δ*, and *spt7Δ* strains under 30 °C and 37 °C conditions..
