# Supplementary material for: Spt7 Deletion Reveals Vulnerabilities in Cryptococcus neoformans Stress Adaptation and Virulence
Source: Microorganisms. 2026 Jan 1;14(1):95. doi: 10.3390/microorganisms14010095 (PMC12844337; doi:10.3390/microorganisms14010095)
Supplement: Supplementary file 1 [file microorganisms-14-00095-s001.zip › Supplementary material.pdf]

Supplementary materials

Supplementary Table S1: Strains used in this study.

Strain	Function	*Species*	Gene	Background	Selective marker	Source
H99O	Wild-type	*C. neoformans*		Clinical		(Janbon *et al.*, 2014)
CJS28	*spt7Δ*	*C. neoformans*	*SPT7*	H99O	*NEO*	This study
KY14	*SPT7* complementation	*C. neoformans*	*SPT7*	CJS28	*NAT*	This study
Mach1	Cloning	*E. coli*		Mach1		Thermo Scientific

Supplementary Table S2: Primers used in this study. SAGA complex gene-specific RT-PCR primers were designed to span exon-exon junctions, ensuring amplification of cDNA rather than genomic DNA.

Fragment / Gene	ID	Sequence
SPT7 – 5' flanking region	UQ5646	5'- GATAAGCTTGATATCGAATTCTGATTTCCCATATATCTCAAG -3'
	UQ5647	5'- TCCAGCTCACATCCTCGCAGCGTTGAAGTATGACCGGTGCAATC -3'
SPT7 – 3' flanking region	UQ5648	5'- CCGTGTTAATACAGATAAACCTAGTGTGAGGGGAAGGTATCT -3'
	UQ5649	5'- GGCCGCTCTAGAACTAGTGGATCCCGAATGTATGTACTTCTT -3'
SPT7 H99O gene overhang with pSDMA25	UQ5655	5'- GGGTACCGGGCCCCCCCTCGATGATTTCCCATATATCTCAAG -3'
	UQ5656	5'- TGGCGGCCGCTCTAGAACTAGCGAATGTATGTACTTCTTTCTG -3'
SPT7 in pSDMA25 sequencing	UQ5562	5'-GCATCACTTACCCATCCTCGA-3'
	UQ5571	5'- TGGCGGCCGCTCTAGAACTAGGCAATCCGCAACCATCAGAGC-3'
	UQ6014	5'-GGGTACCGGGCCCCCCCTCGACAAACAATGGACCTACTTGTA-3'
	UQ5256	5'-CCGTGCGTTTCGTATGTAAGC-3'
SPT7 – *NEO* deletion plasmid sequencing	UQ18	5'- GTAAAACGACGGCCAG -3'
	UQ19	5'- CAGGAAACAGCTATGAC -3'
HFI1 RT-PCR	UQ6258	5'- GTTAAAAGGCGATCATTTACACC -3'
	UQ6259	5'- CGTTGTTGGAGGGAGGA -3'
ADA2 RT-PCR	UQ6260	5'- CAGAGTATCGGGCTATACAAGC -3'
	UQ6261	5'- CTTCGGCAGTTTGCAACTTT -3'
NGG1 RT-PCR	UQ6300	5'- AGTGGATGTGGTTGATTTGG -3'
	UQ6301	5'- TTATTCGGGTCAAACTCTTCG -3'
CHD1 RT-PCR	UQ6262	5'- CTTGCCGATGAAATGGGTCTC -3'
	UQ6263	5'- GAGGGACAACAACAAGGAAAGG -3'
GCN5 RT-PCR	UQ6264	5'- CCGAACAAATCAAGGGTTACG -3'
	UQ6265	5'- TGTCCGCATAAGTCAGAAAGA -3'
SGF11 RT-PCR	UQ6202	5'- ATCAACTGGTGTGGGTAGTG -3'
	UQ6203	5'- ATATCGGTTGGAGGCAATAGG -3'
SGF29 RT-PCR	UQ6266	5'- CAAGTGGCGATGATTGGATTC -3'
	UQ6267	5'- GGGTAGTGTTGTATGTGTTGC -3'
SGF73 RT-PCR	UQ6268	5'- CCGGAAATGAAGAGGGAAGG -3'
	UQ6269	5'- CATGATTACACACCCGTCGAT -3'
SPT3 RT-PCR	UQ6270	5'- GGCACAGGCCCAAGTAAA -3'
	UQ6271	5'- CTCCAAACACAAACATCATCTGC -3'
SPT7 RT-PCR	UQ6302	5'- GTAGATCATCTGGATGGGTTGG -3'

	UQ6303	5’- GGCATGTAGAATGATCTCCTCG -3’
SPT8 RT-PCR	UQ6272	5’- GATGGAACCGTCAGAGAATGG -3’
	UQ6273	5’- GGAAGAGAGTTGGGCTTTGT -3’
SPT20 RT-PCR	UQ6274	5’- GATGGTCCAATGAAGCCATTCC -3’
	UQ6275	5’- GATGGGTGGTTGGATGTCATAAAG -3’
SUS1 RT-PCR	UQ6276	5’- GTTGATGTTACGGTCGATGAG -3’
	UQ6277	5’- AGTAGCTTTTGTATTCGCTCC -3’
TAF5 RT-PCR	UQ6278	5’- CGATGGTGGATGTCAGTGAA -3’
	UQ6279	5’- AGATTATCCAGTTCAGCCTTCC -3’
TAF6 RT-PCR	UQ6280	5’- CGAGTATTGGGCATTTGGTAGA -3’
	UQ6281	5’- GGAGGAGTTGATGCATGTATGG -3’
TAF9 RT-PCR	UQ6222	5’- GAAGCTCCTCCAAGAGATTACC -3’
	UQ6223	5’- GTAGACGGACGAGGTCAAATG -3’
TAF10 RT-PCR	UQ6282	5’- GGTACAATCGTCAATGTGTCC -3’
	UQ6283	5’- ATTAGGTGTGTAGGGTTCATCT -3’
TAF12 RT-PCR	UQ6304	5’- GGCTTTCCGCCAAAGTACC -3’
	UQ6305	5’- GGTTCTGATGTTGGGCGTTATT -3’
TRA1 RT-PCR	UQ6284	5’- TTCTTTCTGTCGGGATTCTCG -3’
	UQ6285	5’- CGCCTTGACGTGTGTAAGATA -3’
UBP8 RT-PCR	UQ6286	5’- GACAAGTTGTCGTGGTCTGC -3’
	UQ6287	5’- TGCTTTGAGAAGCGGATTATGG -3’
ACT1 RT-PCR	UQ482	5’- CCTACAACTCTATCATGAAGTGTGATCTC -3’
	UQ728	5’- TCTGCATACGGTCGGCAATAC -3’
TUB2 RT-PCR	UQ484	5’- AGTCGCTTTTCAAGCGTATCG -3’
	UQ729	5’- GGATTCGGCTTCAGAGAATTCA -3’
GPD1 RT-PCR	UQ486	5’- GTCTCTACTGATTTCGTTGGCACTAC -3’
	UQ730	5’- GTAACCGTACTCATTGTCATACCAGCTA -3'
HHT1 RT-PCR	UQ488	5’- GAAATCCGACGATACCAGAAGTCTAC -3’F
	UQ731	5’- GGAATCGAAGGTCGGTCTTG -3’

Supplementary Table S3: Plasmids used in this study.

Strain	Function	Plasmid backbone	Marker	Source
pJAF1	*NEO* marker	pBluescript SK(-)	*NEO*	Fraser *et al.* (2003)
pSDMA25	Safe Haven 1	pBluescript SK(-)	*NAT*	Arras *et al.* (2015)
pCJS61	*spt7*Δ	pBluescript SK(-)	*NEO*	This study
pKY09	*SPT7* complementation	pSDMA25	*NAT*	This study

Supplementary Table S4A: Proteins consistently differentially expressed in *hfi1*Δ, *spt7*Δ and *spt20*Δ mutants at 30 ˚C.

Protein	*HFI1*		*SPT7*		*SPT20*		Name
	Log2FC	adj.pvalue	Log2FC	adj.pvalue	Log2FC	adj.pvalue	
J9VD88	-0.19471	2.7E-06	-0.17051	3.6E-06	-0.16563	2.68E-08	40S ribosomal protein S3
J9VFJ7	-0.32444	1.08E-07	-0.27176	5.95E-07	-0.25196	3.78E-05	Pescadillo homolog
J9VFZ9	-0.68927	1.85E-05	-1.16043	1.34E-14	-0.71218	4.92E-08	
J9VGK3	0.596977	1.17E-07	0.692626	3.13E-05	0.688992	1.81E-07	Peptide-methionine (R)-S-oxide reductase
J9VHX2	-0.34112	3.37E-10	-0.37549	1.19E-11	-0.24657	4.21E-06	D-lactate dehydrogenase
J9VI58	-0.97285	1.22E-14	-0.18772	3.45E-06	-0.69128	7.19E-10	Succinate dehydrogenase [ubiquinone] cytochrome b small subunit
J9VIR0	-0.19265	2.28E-05	-0.20957	1.66E-05	-0.3339	1.34E-12	alanine--glyoxylate transaminase
J9VIW7	1.022564	1.02E-07	-0.6951	4.99E-05	-0.54272	1.6E-07	Cytochrome c oxidase subunit 6, mitochondrial
J9VK98	-0.45361	1.12E-10	-0.56564	1.08E-10	-0.57228	6.45E-12	Large subunit ribosomal protein L22e
J9VL03	-0.28689	6.54E-05	-0.43528	5.64E-08	-0.62868	2.2E-09	F-type H-transporting ATPase subunit H
J9VM51	-0.43624	4.78E-08	-0.5741	7.95E-06	-1.28067	5.1E-06	ADF-H domain-containing protein
J9VMC4	0.822017	3.77E-08	-0.35356	1.49E-09	-1.06374	1.07E-08	Cytochrome c oxidase subunit 6a
J9VN44	-0.82665	6.27E-07	-0.97305	2.64E-09	-0.8198	3.04E-09	RecF/RecN/SMC N-terminal domain-containing protein
J9VN70	-0.62436	5.6E-12	-0.35509	2.23E-05	-0.61535	1.47E-11	NET1-associated nuclear protein 1 (U3 small nucleolar RNA-associated protein 17)
J9VNA0	-1.54067	5.09E-11	-1.49906	8.31E-11	-0.83144	3.03E-06	Serine/threonine-protein kinase TOR
J9VNS6	-0.73127	5.86E-08	-0.57531	3.89E-06	-0.56151	8.28E-07	Nuclear protein
J9VP48	-0.67328	4.12E-08	-1.02301	7.95E-13	-0.87768	4.4E-13	Endoplasmic reticulum protein
J9VPF2	-0.3744	1.19E-09	-0.35001	3.15E-12	-0.17404	5.34E-05	Small subunit ribosomal protein S10e
J9VPK0	-0.68173	7.43E-07	-0.97382	2.5E-07	-0.98784	1.68E-11	Plasma membrane protein
J9VPZ2	-0.1341	4.85E-06	-0.13512	1.55E-05	-0.17484	3.02E-08	Hydroxymethylglutaryl-CoA synthase
J9VPZ5	-0.30258	6.33E-12	-0.23808	4.16E-08	0.288072	1.29E-11	Mitochondrial carrier
J9VQ25	0.270738	1.57E-08	-0.2609	4.1E-09	-0.26735	8.27E-10	Xaa-Pro dipeptidase
J9VQC3	-0.1759	1.13E-13	-0.17239	1.34E-14	-0.11614	1.95E-07	arginine--tRNA ligase
J9VRA3	-0.50913	2.53E-13	-0.21803	1E-06	-0.23928	3.63E-05	Nascent polypeptide-associated complex subunit alpha

J9VS08	-0.83638	9.64E-08	-1.02904	8.96E-09	-0.80209	1.16E-08	Choline transporter
J9VTU1	-0.97975	4.68E-10	-0.46819	1.19E-05	-1.04895	2.35E-13	Mitochondrial protein
J9VU49	-0.14562	1.35E-07	-0.1901	8.2E-14	-0.22834	2.18E-13	Lysine--tRNA ligase
J9VUQ3	-0.47256	9.21E-13	-0.46348	1.52E-09	-0.33097	9.29E-05	MRS7 family protein
J9VV89	0.301182	1.1E-12	0.210955	9.63E-08	0.192928	2.34E-13	Transaldolase
J9VWM3	-0.53485	2.41E-06	-0.69293	5.09E-06	-0.69258	1.14E-07	Ubiquinone biosynthesis monooxygenase COQ6, mitochondrial
J9VWW7	-0.65953	2.34E-10	-0.79275	6.38E-14	-0.51526	1.52E-05	ATPase
J9VXB7	-0.65042	3.07E-05	-0.91949	9.29E-07	-0.73948	3.01E-06	Protein transporter SEC61 subunit alpha
J9VXH1	-0.62291	2.62E-05	-0.65706	5.13E-07	-0.76213	9.32E-07	Ribonucleoside-diphosphate reductase subunit M2
J9VXI8	-0.22105	1.91E-08	0.203868	2.82E-09	0.218099	2.81E-12	Sulfite reductase (NADPH) flavoprotein alpha-component
J9VXN1	-0.66304	2.91E-09	-0.92293	5.16E-14	-0.62907	1.41E-05	
J9VY09	0.163082	3.78E-05	-0.28758	1.29E-12	-0.19476	4.57E-08	Succinate--CoA ligase [ADP-forming] subunit alpha, mitochondrial
J9VY76	-1.18177	1.14E-09	-0.95065	1.84E-08	-1.16876	2.38E-06	Long-chain fatty acid transporter
J9VYF7	0.681104	4.42E-07	-0.80005	1.95E-06	-0.44783	4E-05	serine C-palmitoyltransferase
J9VZA3	-0.48785	8.83E-07	-0.2303	7.98E-11	-0.32831	3.63E-05	Non-structural maintenance of chromosomes element 1 homolog
J9VZI7	-0.16401	6.3E-07	-0.26706	4.76E-09	-0.21012	7.95E-08	40S ribosomal protein S7
J9VZT6	-0.44635	1.21E-11	-0.47122	9.22E-10	-0.40613	2.24E-07	40S ribosomal protein S26
J9W0I6	-0.36931	1.57E-08	-0.40051	8.28E-10	-0.29568	2.02E-06	Large subunit ribosomal protein L13
J9W140	2.064079	8.51E-06	2.139114	1.23E-05	1.085668	2.77E-07	
J9W1B7	0.333291	4.61E-05	-0.86235	8.73E-14	-0.65104	2.05E-10	U6 snRNA-associated Sm-like protein LSm2
J9W3C1	-0.8011	9.68E-05	-0.83072	1.14E-09	-0.91477	9.3E-14	Mitochondrial splicing suppressor
Q059G5	-0.83093	5.42E-12	-0.75747	1.62E-11	-0.67932	1.59E-12	Trehalase
T2BQ21	-0.5122	5.74E-11	-0.31486	1.49E-07	-0.84809	4.7E-13	CMGC/CDK/CDK5 protein kinase

Supplementary Table S4B: Proteins consistently differentially expressed in *hfi1*Δ, *spt7*Δ and *spt20*Δ mutants at 37 ˚C.

ID	*HFI1*		*SPT7*		*SPT20*		Name
	Log2FC	adj.pvalue	Log2FC	adj.pvalue	Log2FC	adj.pvalue	
J9VDW0	-0.79223	2.49E-06	-1.61432	2.38E-09	0.495696	1.66E-05	MICOS complex subunit
J9VEY5	-0.22759	3.1E-05	-0.40514	5.37E-11	-0.2076	2.03E-08	Small ribosomal subunit protein mS29
J9VF89	-0.7713	2.88E-08	-0.74667	1.4E-10	-0.31566	1.44E-05	U3 small nucleolar RNA-associated protein 18
J9VFW4	0.327724	4.94E-13	-0.22592	4.67E-07	-0.34849	4.73E-10	Elongation factor 3
J9VFX5	0.23239	1.59E-06	0.213105	5.72E-06	0.200343	1.33E-06	Chlorophyll synthesis pathway protein BchC
J9VGF3	0.261635	6.14E-06	0.437535	2.68E-12	-0.28769	8.93E-08	4-nitrophenylphosphatase
J9VGW2	2.13361	4.64E-07	0.601545	5.24E-08	-1.61637	7.18E-15	Endoplasmic reticulum protein
J9VH51	0.668072	3.12E-05	0.693964	1.05E-08	0.455972	3.65E-10	Squalene synthase
J9VHR9	0.160314	2.02E-05	-0.28838	2.29E-07	-0.21888	3.14E-08	Kynurenine aminotransferase
J9VI22	0.448829	3.08E-10	0.394163	2E-11	-0.30874	8.2E-06	Short-chain dehydrogenase
J9VJK6	0.263681	7.38E-13	0.274228	7.85E-10	-0.22178	1.34E-12	Histidine biosynthesis trifunctional protein
J9VK98	-0.62401	9.13E-10	-0.52046	2.89E-09	-0.56564	1.08E-10	Large subunit ribosomal protein L22e
J9VKY5	-0.29861	3.63E-14	-0.26408	1.15E-06	-0.18721	1.63E-07	Glutaredoxin
J9VLJ9	-0.30945	6.65E-10	-0.28428	4.56E-13	0.31909	3.53E-07	Superoxide dismutase [Cu-Zn]
J9VML6	0.306672	9.16E-11	0.259957	1.65E-07	0.303041	4.36E-10	Nucleosome assembly protein 1-like 1
J9VN50	0.20542	3.5E-11	0.225342	1.82E-09	0.184873	8.48E-07	NAD dependent epimerase/dehydratase
J9VPZ6	0.658102	6.46E-13	0.582465	3.62E-09	0.606084	6.02E-08	Ubiquitin carboxyl-terminal hydrolase
J9VQ69	0.406325	1.16E-14	0.276399	6.3E-08	0.313651	4.75E-09	RRM domain-containing protein
J9VQ78	-1.0461	1.42E-09	-0.80554	3.2E-07	-0.78519	1.21E-13	ATP-binding cassette transporter
J9VQB8	-0.47364	2.2E-10	-0.44763	2.98E-14	-0.49973	2.61E-13	Aminomethyltransferase
J9VQQ8	0.318547	5.01E-05	-0.38892	4.84E-05	-0.29807	1.2E-05	Translation machinery-associated protein 20
J9VR17	0.604141	3.13E-07	0.498684	1.23E-07	-0.55964	5.49E-06	Sugar phosphate phosphatase
J9VR33	-0.48775	6.87E-09	-0.76217	3.15E-14	-0.49805	5.57E-11	60S ribosomal protein L36
J9VR76	0.241601	5.71E-08	0.410104	2.67E-09	-0.18456	8.39E-05	Voltage-gated potassium channel protein beta-2 subunit
J9VRA3	-0.28775	6.58E-06	-0.87222	2.08E-12	-0.21803	1E-06	Nascent polypeptide-associated complex subunit alpha
J9VST6	0.494027	7.34E-13	-0.25191	9.25E-05	-0.31429	1.18E-09	KH domain-containing protein

J9VT09	-0.37223	1.77E-05	-0.46095	1.17E-07	-0.17039	6E-05	Eukaryotic translation initiation factor 6
J9VT31	-0.21428	8.2E-09	-0.30585	1.3E-11	0.187011	8.23E-08	Triosephosphate isomerase
J9VTE3	-0.37192	3.97E-08	-0.45976	4E-12	-0.3767	7.18E-15	Small subunit ribosomal protein S17
J9VTJ1	-0.28143	2.77E-05	-0.53694	1.6E-13	-0.48253	1.52E-07	Eukaryotic translation initiation factor 3 subunit K
J9VTJ6	-0.63283	1.92E-06	-0.7694	3.87E-05	0.450914	4.19E-05	V-type proton ATPase subunit a
J9VUV2	-0.34796	8.24E-07	-0.61989	7.73E-15	-0.35471	5.44E-07	Large subunit ribosomal protein L2
J9VV32	0.913905	1.27E-13	0.88003	8.61E-12	-0.98951	6.71E-12	GST N-terminal domain-containing protein
J9VWJ8	-0.59638	3.04E-15	-0.38408	1.8E-07	-0.27151	6.15E-05	ATP synthase subunit 4
J9VXA7	1.189043	1.3E-08	0.774461	2.17E-05	-0.44701	2.25E-06	Translocation protein SEC62
J9VXE8	-0.28828	7.27E-05	-0.57939	1.88E-14	-0.29626	1.27E-05	Large subunit ribosomal protein L3
J9VXN1	-0.31596	4.63E-05	-0.80814	6.72E-10	-0.92293	5.16E-14	
J9VYI2	0.192422	5.27E-06	-0.29879	5.94E-08	-0.17046	1.03E-05	Clustered mitochondria protein homolog
J9VZT6	-0.46864	1.07E-13	-0.60761	4.42E-14	-0.47122	9.22E-10	40S ribosomal protein S26
J9W0F1	-0.71965	7.3E-07	-0.87519	7.77E-09	-0.64503	9.84E-09	Cytoplasmic protein
J9W0I6	-0.58538	3.23E-11	-0.58597	2.25E-12	-0.40051	8.28E-10	Large subunit ribosomal protein L13
J9W0J1	0.436536	2.25E-09	0.452921	7.26E-10	-0.33923	2.31E-08	Enoyl reductase
J9W1B7	-0.94141	3E-13	-1.13231	1.15E-14	-0.86235	8.73E-14	U6 snRNA-associated Sm-like protein LSm2
J9W1J0	1.512333	1.4E-08	1.38266	4.81E-06	0.186734	6.05E-05	Kexin
J9W1J2	0.240682	1.22E-07	-0.35503	3.04E-10	-0.25902	2.4E-12	Pre-mRNA-processing factor 19
J9W1W1	-0.49586	2.46E-10	-0.89278	5.04E-06	-0.32486	9.58E-07	Aspartate-tRNA ligase
J9W2U5	-1.08592	1.82E-07	-0.99044	1.03E-10	-0.56994	2.24E-11	Large subunit ribosomal protein L32e
Q56R42	0.337138	2.82E-11	-0.28913	5.14E-08	-0.22831	1.26E-05	Mitogen-activated protein kinase HOG1

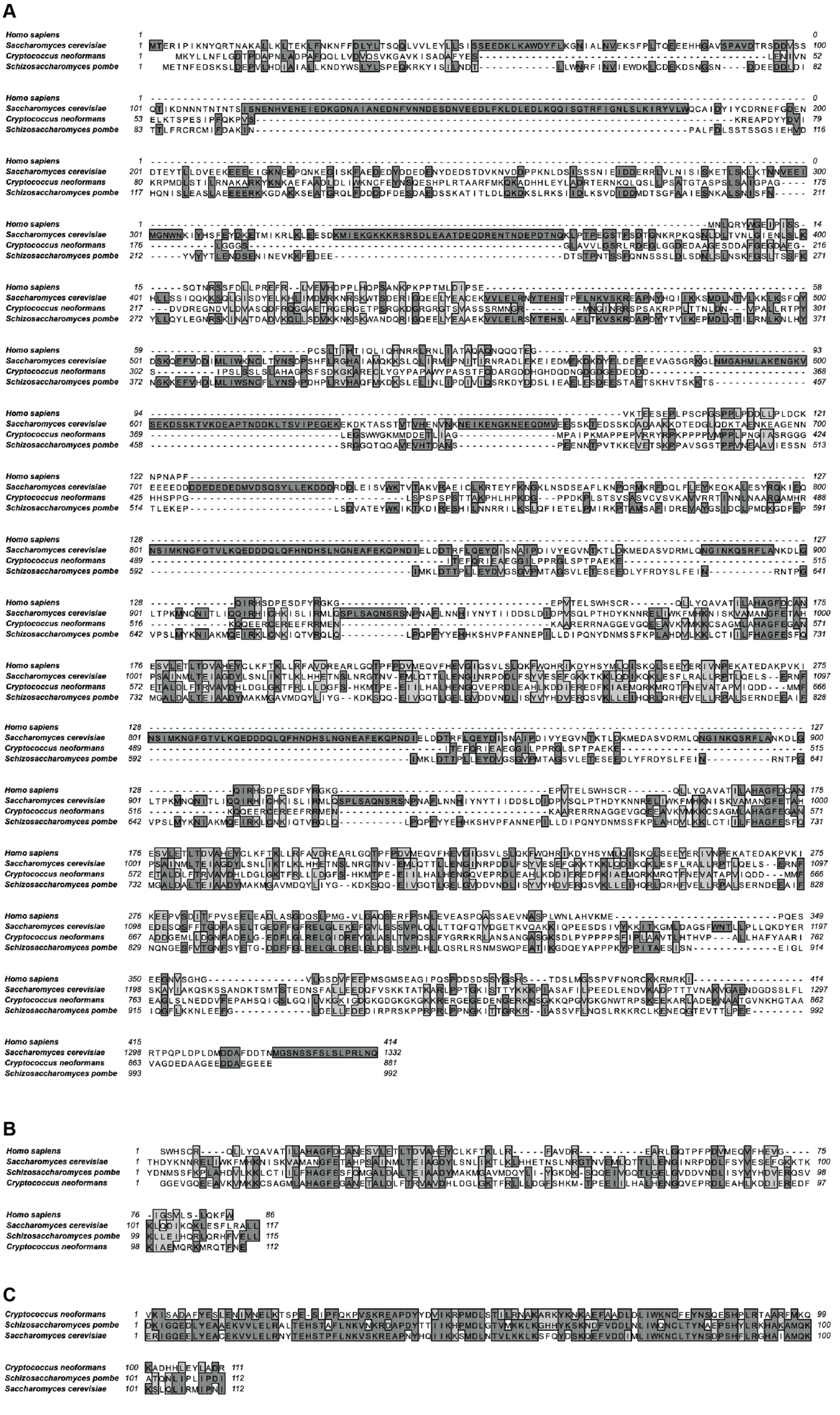

Supplementary Figure S1: A) Protein alignments of full length Spt7 from *S. cerevisiae*, *S. pombe*, *C. neoformans* and *H. sapiens*. B) Protein alignments of HFD of Spt7 from *S. cerevisiae*, *S. pombe*, *C. neoformans* and *H. sapiens*. C) Protein alignments of Bromo domain of Spt7 from *S. cerevisiae*, *S. pombe* and *C. neoformans*
